# Sleep continuity: a new metric to quantify disrupted hypnograms in non-sedated intensive care unit patients

**DOI:** 10.1186/s13054-014-0628-4

**Published:** 2014-11-25

**Authors:** Xavier Drouot, Agathe Bridoux, Arnaud Wilfrid Thille, Ferran Roche-Campo, Ana Cordoba-Izquierdo, Sandrine Katsahian, Laurent Brochard, Marie-Pia d’Ortho

**Affiliations:** CHU de Poitiers, Service d’Explorations Fonctionnelles, de Physiologie Respiratoire et de l’Exercice et Service de Neurophysiologie Clinique, Poitiers, F-86021 France; Univ Poitiers, Faculté de Medecine et de Pharmacie, Poitiers, France; INSERM, CIC 1402, Poitiers, France; EA 4391, Université Paris Est, Créteil, F-94010 France; APHP, groupe Henri Mondor – Albert Chenevier, Service de Physiologie, Creteil, France; CHU de Poitiers, Service de réanimation médicale, Poitiers, France; Hospital Sant Pau, Servei de Medicina Intensiva, Barcelona, Spain; Hospital Universitari de Bellvitge, Servei de Pneumologia, L’Hospitalet de Llobregat, Barcelona, Spain; APHP, Groupe Henri Mondor - Albert Chenevier, Unité de Recherche Clinique - Santé Publique, Créteil, France; St Michael’s Hospital, Toronto, Canada; University of Toronto, Toronto, Canada; AP-HP, Groupe Bichat-Claude Bernard, Service de Physiologie, Paris, France; Univ Denis-Diderot-Paris 7, Paris, France

## Abstract

**Introduction:**

Sleep in intensive care unit (ICU) patients is severely altered. In a large proportion of critically ill patients, conventional sleep electroencephalogram (EEG) patterns are replaced by atypical sleep. On the other hand, some non-sedated patients can display usual sleep EEG patterns. In the latter, sleep is highly fragmented and disrupted and conventional rules may not be optimal. We sought to determine whether sleep continuity could be a useful metric to quantify the amount of sleep with recuperative function in critically ill patients with usual sleep EEG features.

**Methods:**

We retrospectively reanalyzed polysomnographies recorded in non-sedated critically ill patients requiring non-invasive ventilation (NIV) for acute hypercapnic respiratory failure. Using conventional rules, we built two-state hypnograms (sleep and wake) and identified all sleep episodes. The percentage of time spent in sleep bouts (<10 minutes), short naps (>10 and <30 minutes) and long naps (>30 minutes) was used to describe sleep continuity. In a first study, we compared these measures regarding good (NIV success) or poor outcome (NIV failure). In a second study performed on a different patient group, we compared these measurements during NIV and during spontaneous breathing.

**Results:**

While fragmentation indices were similar in the two groups, the percentage of total sleep time spent in short naps was higher and the percentage of sleep time spent in sleep bouts was lower in patients with successful NIV. The percentage of total sleep time spent in long naps was higher and the percentage of sleep time spent in sleep bouts was lower during NIV than during spontaneous breathing; the level of reproducibility of sleep continuity measures between scorers was high.

**Conclusions:**

Sleep continuity measurements could constitute a clinically relevant and reproducible assessment of sleep disruption in non-sedated ICU patients with usual sleep EEG.

**Electronic supplementary material:**

The online version of this article (doi:10.1186/s13054-014-0628-4) contains supplementary material, which is available to authorized users.

## Introduction

Most patients in intensive care units (ICUs) experience severe sleep disruptions. These alterations include decreased total sleep time and slow wave sleep, decreased rapid eye movement (REM) sleep and marked fragmentation [1-6]. They may have clinical consequences in critically ill patients, but few studies have linked sleep disruptions and outcomes [7].

Quantification of sleep in ICU patients is a major concern since sleep is highly altered and very different from sleep in non-ICU patients, that is, patients with obstructive sleep apnea syndrome. In about one third of ICU patients, the conventional scoring rules of the American Academy of Sleep Medicine (AASM) [8,9] are difficult to use because of altered sleep and wake electroencephalogram (EEG) patterns [10,11] and alternative methods have been recently proposed [12-14].

When applicable, conventional sleep parameters show high inter-individual variations in ICU populations [4,15]. In addition, patients in ICU display abnormal sleep/wake organization with non-consolidated polyphasic sleep. Patients often spend as much as 50% of their sleep during daytime naps [1,4,15], in contrast to the consolidated nocturnal sleep observed in ambulatory patients. Traditional assessments of sleep in this patient population may capture only the gross features of severely disorganized sleep architecture. The relationship between sleep disruptions in ICU and clinical consequences is difficult to establish, prompting a search for additional parameters that could enhance sleep quality assessment.

Based on Bonnet’s sleep continuity theory [16], which posits that at least 10 minutes of uninterrupted sleep are needed to serve a recuperative function, several authors have deemed quantification of sleep continuity to be of interest. Two studies showed less continuous sleep in patients with sleep-disordered breathing compared to healthy subjects, whereas the usual parameters were not statistically different between groups [17,18]. Regarding the disrupted hypnograms of critically ill patients, we tried to find and develop a simple and pertinent measure of sleep continuity.

Since sleep deprivation has been shown to reduce inspiratory muscle endurance in healthy subjects [19], we postulated that lack of restorative sleep might impact respiratory functions in ICU patients. Our hypothesis was that sleep episodes lasting less than 10 minutes would be less restorative than sleep episodes lasting more than 10 minutes. We studied sleep in non-sedated ICU patients with hypercapnic respiratory failure treated with non-invasive ventilation (NIV) during several days. We then used NIV outcome as a ‘measure of performance’, that could indirectly investigate sleep’s restorative function.

Our aim was to determine whether or not a sizable proportion of sleep spent in long episodes was associated with NIV success. We also compared sleep continuity during NIV and during spontaneous breathing in a second group and tested the inter-scorer reproducibility of these measures.

## Methods

### Patients

We included adults who were admitted to a medical ICU for acute hypercapnic respiratory failure, treated with NIV for at least two days and enrolled in two previously published studies [7,20]. Acute hypercapnic respiratory failure was defined by respiratory rate >22 breath/minutes, pH <7.35 and partial pressure of CO_2_ (PaCO2) >45. We did not include patients needing endotracheal intubation within the first 48 hours, patients with encephalopathy (defined as a Richmond Agitation-Sedation Scale score ≤1) or with documented obstructive sleep apnea and patients who rapidly improved and could be weaned from NIV within 48 hours. Exclusion criteria included occurrence of encephalopathy, sedative, opioid or neuroleptic drugs administered within the last 48 hours, neurologic or psychiatric disease and hemodynamic instability. We, therefore, focused on a very homogeneous subgroup with acute hypercapnic respiratory failure treated with NIV for more than 48 hours.

#### Polysomnographies

All polysomnography (PSG) recordings were obtained using an S700 recorder (Embla, Denver, CO, USA) for 17 hours (4 pm to 9 am) with six EEG channels (F3-A2, F4-A1, C4-A1, C3-A2, O2-A1 and O1-A2), a chin electromyogram, two electrooculograms and submental electromyogram. We began PSG in the afternoon because morning ablutions, nursing care, medical examination and respiratory therapy could disconnect electrodes, and because patients are solicited and likely to be awake during this morning period. The sticking of captors started at 2 pm, followed by bio-calibration procedures with patients maintained awake, and recordings started at 4 pm.

No patients received sedatives or analgesics during, and 48 hours before, PSG. All patients and/or next of kin gave written informed consent before study inclusion. All studies were approved by the Ethics Committee of the French Society of Intensive Care Medicine.

#### Conventional sleep metrics

Conventional sleep parameters included total sleep time, non-REM and REM sleep duration, and number of arousals and awakenings per hour of sleep.

#### Sleep continuity

Sleep stages and awakenings were scored using the AASM scoring rules and criteria [8,9]. We only included PSG displaying usual sleep/wake EEG patterns. Patients displaying atypical sleep and pathological wakefulness [13] were excluded from analysis for three reasons: First, atypical sleep might be a particular kind of sleep, clearly distinct from sleep with usual EEG patterns, and which has already been associated with poor outcome [7]. Second, we wished to validate sleep continuity on a homogenous set of polysomnographies. Third, atypical sleep can be easily identified based on EEG criteria and is thought to occur in one third of patients [13,14]. The main idea of our study was to search for a new tool to quantify sleep in the other two thirds of ICU patients, who display usual sleep EEG patterns but with disrupted hypnograms.

**Figure 1:**
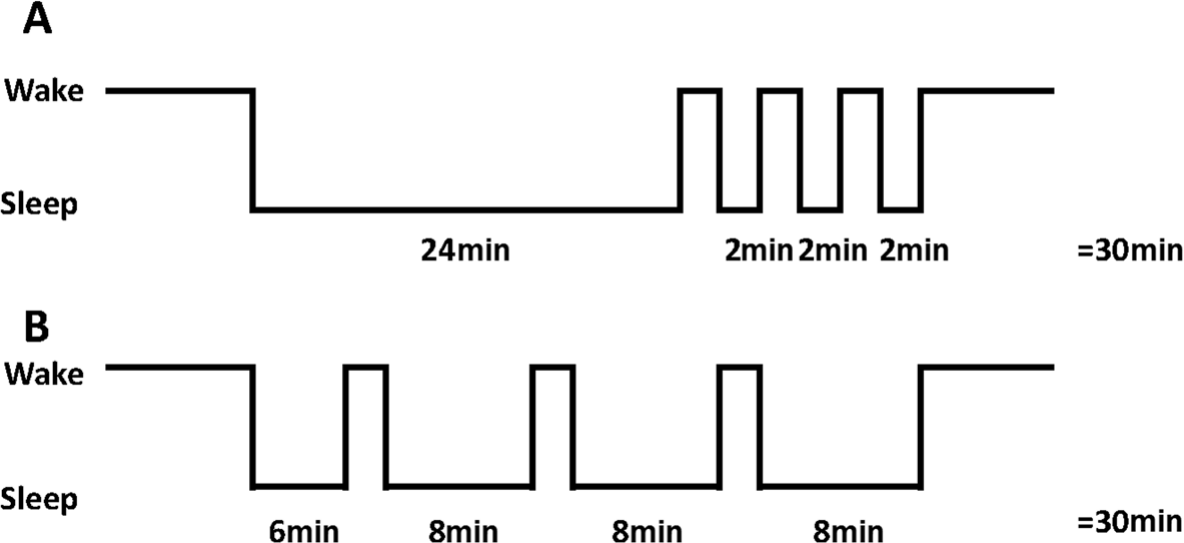
**Illustration of the potential superiority of sleep continuity measures over conventional sleep analysis.** The two hypnograms are similar as concerns the amount of sleep and awakening index. Only sleep continuity measures such as time spent in short naps of 10- to 30-minute duration (24 minutes (80% of sleep time) in hypnogram **A** versus 0 minutes [0%] in hypnogram **B)** or time spent in bouts of less than 10 minutes (6 minutes (20%) in A versus 30 minutes (100%) in B) could differentiate the two hypnograms.

We also excluded patients who slept less than one hour because they could be easily considered as sleep-deprived and because we thought that a minimal amount of sleep was required to analyze sleep continuity. Our hypothesis was that, for instance, one 24-minute continuous sleep episode would be more restorative and clinically more profitable than three successive 8-minute episodes (Figure 1).

We then transformed conventional hypnograms into two-state hypnograms and proceeded as previously reported [17]. A sleep episode began with a change from wakefulness to any stage of sleep and continued until there was a change from any sleep stage to wakefulness. For each PSG, we identified all sleep episodes and their duration.

The parameters used to assess sleep continuity were as follows. We calculated the time spent (expressed as percentage of total sleep time) in sleep bouts (defined as sleep episodes lasting less than 10 minutes), in short naps (defined as sleep episodes lasting 10 to 30 minutes) and in long naps (defined as sleep episodes lasting more than 30 minutes). We chose thresholds of 10 and 30 minutes because many studies have shown that naps lasting 10 to 30 minutes are the most restorative on fatigue and performance impairments induced by sleep deprivation [21-24].

High sleep continuity could be defined as the association of a high amount of sleep spent in naps and a low amount of sleep spent in sleep bouts.

#### Study 1: Clinical relevance of sleep continuity measures

The main objective of this work was to determine whether sleep quality within the first 72 hours after admission could predict NIV failure in patients with acute respiratory failure treated with NIV. Since sleep EEG could be unusual in ICU patients (precluding use of Rechtschaffen and Kales rules), the first step was to identify if wake/sleep EEG pattern could predict NIV failure [7]. The second step planned in this prospective work was to search for sleep parameters that could predict NIV failure in patients with usual sleep EEG patterns (in whom standard Rechtschaffen and Kales rules could be used).

We analyzed hypnograms of the 16 patients with normal sleep/wake EEG patterns (that is, absence of atypical sleep) and with total sleep time (TST) >60 minutes. Patients were classified into NIV success (n = 10) group or NIV failure group (n = 6) regarding outcome. NIV failure was defined by death or endotracheal intubation or need for at least four hours of NIV per day on the sixth day of NIV. We compared sleep continuity parameters and conventional analysis between the two groups.

#### Study 2: Comparison of sleep continuity during NIV and during spontaneous breathing

In this study involving 24 non-sedated ICU patients with acute hypercapnic respiratory failure [20] and with normal sleep/wake EEG patterns, we retrospectively measured and compared sleep time spent in sleep bouts, short naps and long naps calculated when patients were spontaneously breathing and when they were under NIV with pressure support. We wished to determine whether sleep continuity measures would yield results similar to those obtained in conventional analysis.

To perform an advanced evaluation of sleep continuity, we added a statistical validation and calculated the interscorer reproducibility of this new measurement tool. Two trained sleep specialists (XD and AB) independently scored all polysomnographies (n = 24) performed in the study by Cordoba-Izquierdo [20]. Sleep continuity was calculated on the entire PSG. Agreement between scorers was assessed by calculating Cohen kappa for hypnograms and intraclass coefficient for sleep continuity.

### Statistics

Continuous variables were expressed as median [25th to 75th percentile] and were compared using the non-parametric unpaired test (Mann–Whitney test) or the non-parametric paired test (Wilcoxon rank test). Statistical significance was set at *P* <0.05.

The Cohen kappa coefficient is the gold standard to assess agreement of qualitative data and is often used for pairwise comparison of sleep scoring [25]. A kappa coefficient >0.8 is indicative of excellent agreement and of good and reasonable agreement when it is between 0.61 to 0.80 and 0.41 to 0.60, respectively.

To measure interscorer agreement for continuity parameters, we calculated the intraclass coefficient (ICC), an equivalent of kappa coefficient for quantitative data. For each patient, sleep continuity parameters were calculated from the two scorings. ICCs were calculated for each continuity parameter. An ICC >0.8 is indicative of good agreement between the two datasets.

## Results

### Study 1: Clinical relevance of sleep continuity

#### Patients

**Figure 2:**
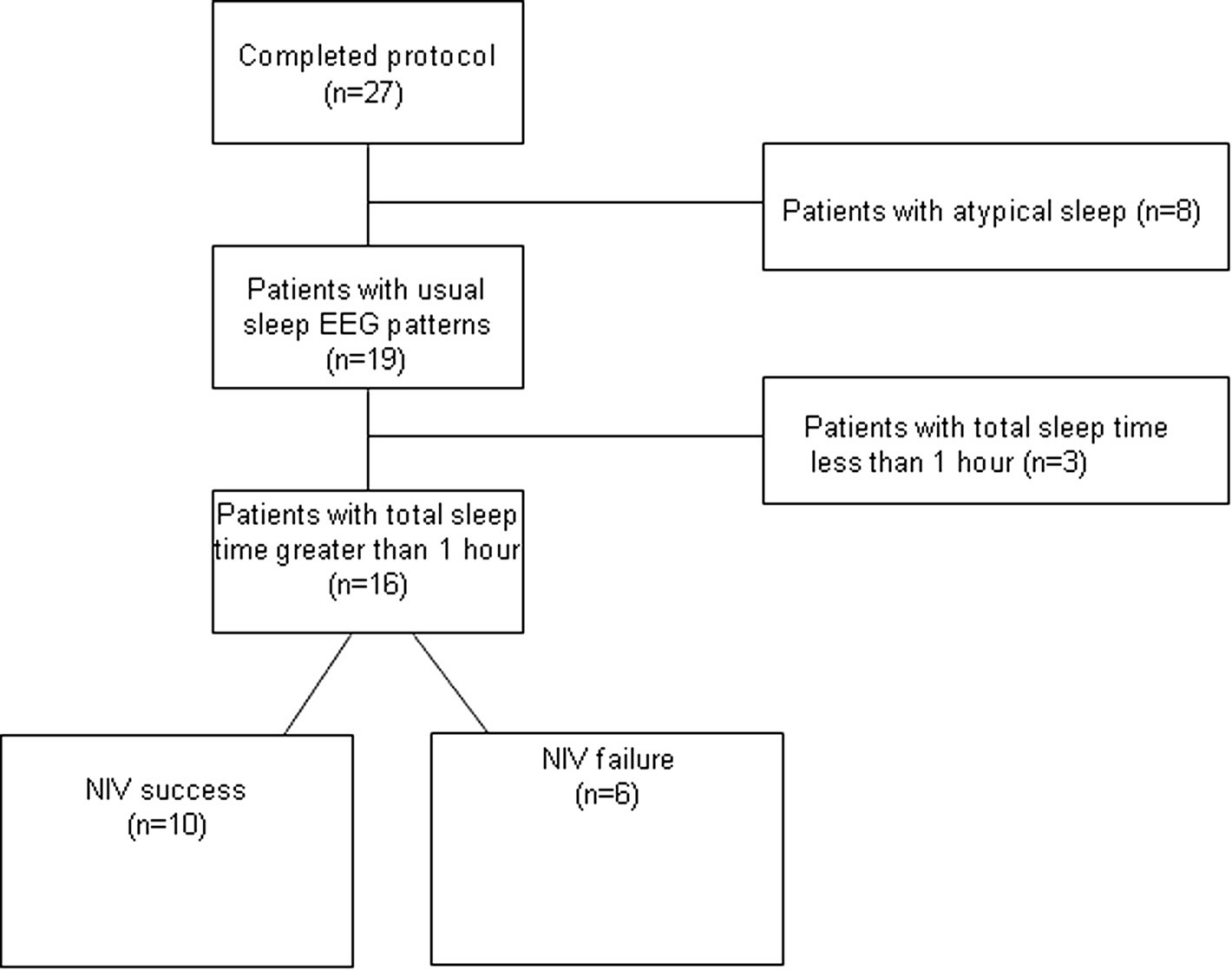
**Study 1 flow chart.** The diagram illustrates patient selection in study 1. NIV: non-invasive ventilation.

Clinical characteristics and respiratory parameters on the study day were not statistically different between patients with NIV failure (n = 6) and those with NIV success (n = 10) (Table 1). The flow chart is illustrated in Figure 2.

**Table 1:** **Clinical characteristics and respiratory parameters in study 1 patients**

**Characteristics**	**NIV success**	**NIV failure**	***P*** **value**
	**number = 10 (62%)**	**number = 6 (38%)**	
Age, years	79 (60 to 84)	84 (82 to 87)	0.1
Female, number (%)	3 (30%)	6 (100%)	
Known COPD^a^	7 (70%)	4 (67%)	
Known heart failure^a^	3 (30%)	4 (67%)	
SAPS II score (at admission)	41 (34 to 45)	39 (35 to 43)	0.9
pH	7.38 (7.35 to 7.41)	7.31 (7.30 to 7.31)	0.1
PO_2_/FiO_2_, mm Hg	269 (199 to 340)	340 (300 to 358)	0.3
PCO_2_, mm Hg	69 (46 to 70)	66 (62 to 77)	0.5
Bicarbonates, mmol/L	38 (29 to 40)	36 (31 to 41)	0.5
NIV duration prior PSG (hours)	12 (8 to 18)	17 (14 to 20)	0.4

^a^Known at admission. *P* values from the Mann–Whitney test. Values are given in median (25th to 75th percentiles). COPD: chronic obstructive pulmonary disease; NIV: non-invasive ventilation; PSG: polysomnography; SAPS: Simplified Acute Physiology Score.

#### Conventional sleep metrics

Traditional sleep parameters were not statistically different between patients with NIV failure and those with NIV success (Table 2).

**Table 2:** **Sleep parameters in patients with NIV success and NIV failure (study 1)**

**Parameters**	**NIV Success (number = 10)**	**NIV failure (number = 6)**	***P*** **value**
TST (minutes)	423 (359 to 458)	328 (274 to 422)	0.3
Stage N1 (%)	16 (12 to 20)	17 (9 to 23)	0.6
Stage N2 (%)	45 (35 to 50)	61 (55 to 63)	0.2
Stage N3 (%)	12 (8 to 17)	13 (6 to 16)	0.6
REM (%)	11 (6 to 14)	3 (3 to 6)	0.1
Fragmentation (n/h)	27 (20 to 30)	36 (31 to 43)	0.1

Fragmentation, number of arousals and awakenings per hour of sleep; values are median (25th to 75th percentiles). *P* values from the Mann–Whitney test. NIV: non-invasive ventilation; REM: rapid eye movement; TST: total sleep time.

#### Hypnogram quantification

**Figure 3:**
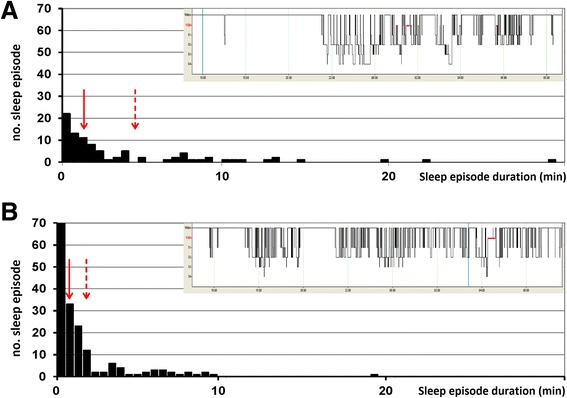
**Hypnogram and sleep episode distribution in two representative patients. A)** Frequency of sleep episode duration in a patient with high sleep continuity. Note that the number of sleep bouts is low. This patient spent most of his sleep time in naps lasting more than 10 minutes. Total sleep was 349 minutes. Inset: conventional hypnograms from 4 pm to 9 am. Arrow: median value (1.5 minutes); Dash arrow: 75th percentile value (4.75 minutes). **B)** Frequency of sleep episode duration in a patient with low sleep continuity. The number of bouts is high and there is only one nap lasting more than 10 minutes. This patient spent most of his sleep in bouts shorter than 10 minutes. Total sleep was 326 minutes. Inset: conventional hypnograms from 4 pm to 9 am. Arrow: median value (one minute); Dash arrow: 75th percentile value (two minutes).

Figure 3 illustrates sleep episode distribution in two representative patients.

**Figure 4:**
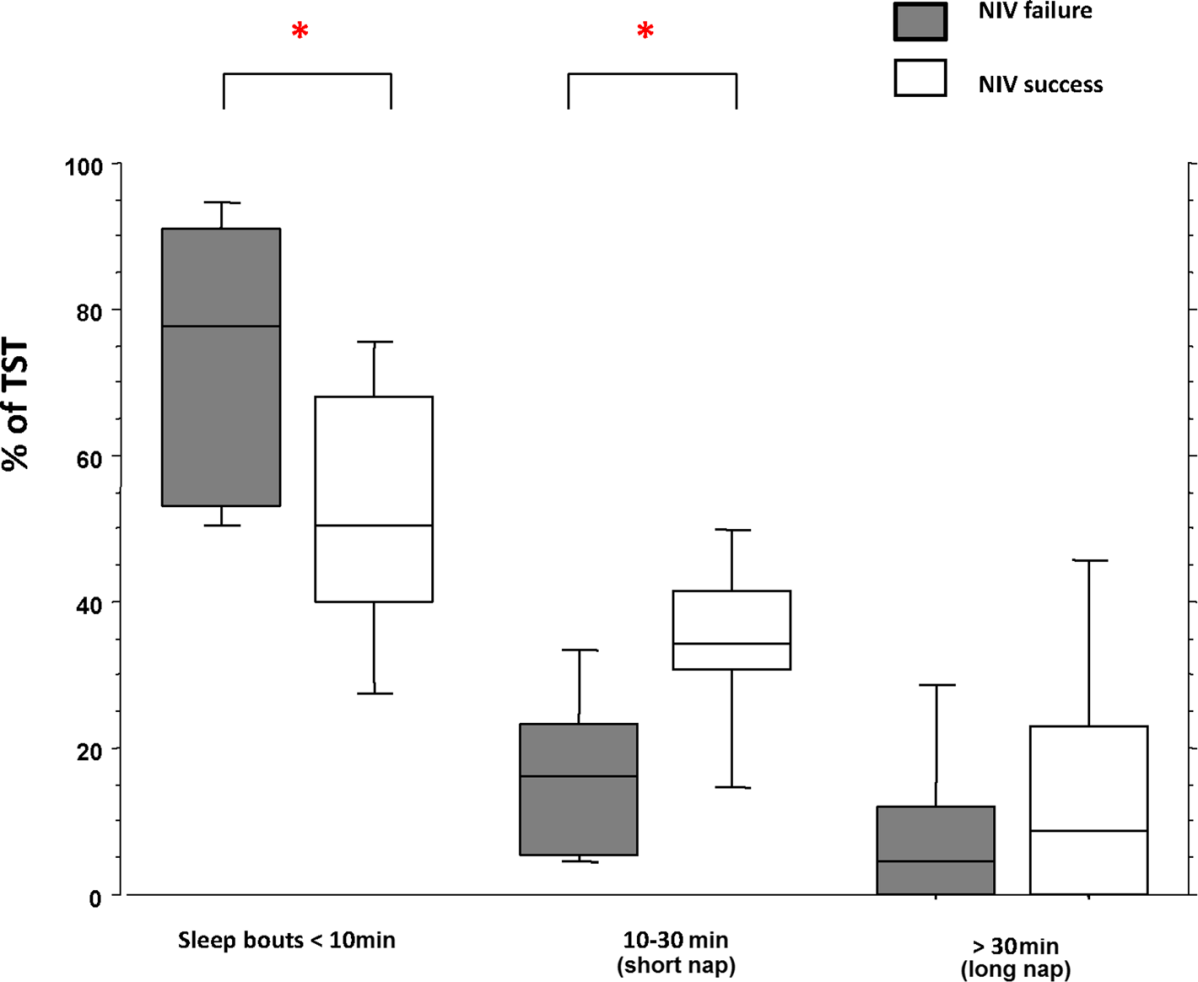
**Clinical relevance of sleep continuity: sleep time spent in sleep bouts, short naps and long naps in patients with NIV success and NIV failure.** Average sleep time (expressed as % of total sleep time) spent in sleep bouts lasting less than 10 minutes, short naps (10 to 30 minutes) and long naps (>30 minutes) in the NIV failure group (gray boxes) and in NIV success patients (white boxes). Bottom and top of boxes are 25th and 75th percentiles, respectively, middle lines are median; lower and upper whiskers are 10th and 90th percentile, respectively; * *P* <0.05, Mann Whitney test between groups. NIV: non-invasive ventilation.

Sleep time spent in sleep bouts (<10 minutes), expressed as a percentage of TST, was significantly lower in patients with successful NIV than in patients who failed (54% (45 to 69) versus 84% (58 to 99), *P* <0.05, respectively). Sleep time spent in short naps lasting between 10 and 30 minutes, expressed as a percentage of TST, was significantly higher in patients with successful NIV than in patients who failed (32% (20 to 38) versus 16% (8 to 23), *P* <0.05, respectively, Mann Whitney test) (Figure 4). Finally, sleep time spent in long naps, lasting more than 30 minutes, expressed as a percentage of TST, did not significantly differ between the two groups (9% (0 to 25) versus 5% (0 to 11), *P* >0.05).

### Study 2: Comparison of sleep continuity during NIV and during spontaneous breathing

**Figure 5:**
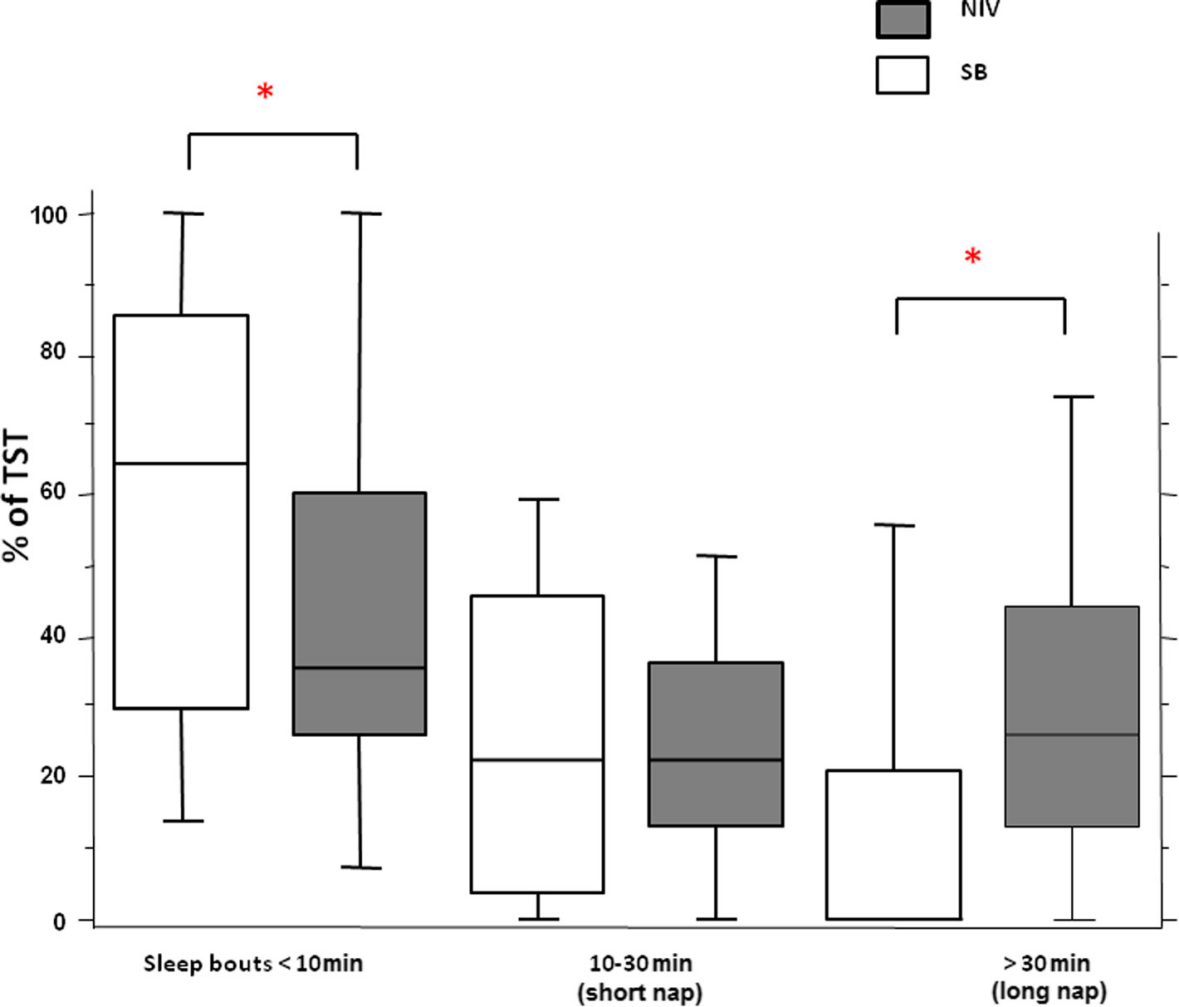
**Effect of NIV on sleep continuity.** Sleep time spent in sleep bouts, short naps and long naps during NIV and spontaneous breathing. Average sleep time (expressed as % of total sleep time) spent in sleep bouts lasting less than 10 minutes, short naps (10 to 30 minutes) and long naps (>30 minutes) during NIV (white boxes) and during spontaneous breathing (SB) (gray boxes) in 24 patients. Bottom and top of boxes are 25th and 75th percentiles, respectively, middle lines are median, lower and upper whiskers are 10th and 90th percentile, respectively; * *P* <0.05, Wilcoxon signed Rank Test. NIV, non-invasive ventilation.

The flow chart of the study is illustrated in Additional file 1. Clinical characteristics and respiratory parameters at the study day are presented in Table 3. In this study sample, conventional sleep analysis revealed a better sleep quality during NIV (less fragmentation and a more normal percentage of slow wave sleep, REM sleep, and N1 sleep) than sleep during spontaneous ventilation (Table 4) [20]. The percentage of sleep time spent in sleep bouts was significantly lower during NIV (35% (27 to 59) versus 65% (30 to 86), *P* <0.05, Wilcoxon rank test). The percentage of sleep time spent in short naps was not statistically different between NIV and spontaneous ventilation, respectively (22% (13 to 35) versus 22% (7 to 45), *P* >0.05). The percentage of sleep time spent in long naps was significantly higher (26% (13 to 43) versus 0% (0 to 20), *P* < 0.006) when patients slept under NIV compared to spontaneous ventilation (Figure 5).

**Table 3:** **Clinical characteristics of the 24 patients included in study 2**

**Characteristics**	**median (25** ^**th**^ **-75** ^**th**^ **)**
Age, years	69 (65 to 77)
Female sex, number (%)	10 (42)
BMI (kg/m^2^)	29 (20 to 37)
Known COPD^a^, number (%)	11 (46)
Other underlying chronic respiratory disease number (%)	6 (25)
SAPS II score (at admission)	30 (25 to 35)
pH	7.37 (7.33 to 7.40)
PCO_2,_ mmHg	64 (57 to 71)
Bicarbonate, mmol/L	38 (33 to 43)
Previous time on NIV, hours	18 (12 to 22)

^a^Known at admission. Values are given in median (25th to 75th percentiles). *P* values from the Mann–Whitney test. BMI: body mass index; COPD: chronic obstructive pulmonary disease; NIV: non-invasive ventilation; SAPS: Simplified Acute Physiology Score.

**Table 4:** **Sleep parameters during NIV and during spontaneous breathing in study 2 patients**

**Parameters**	**During NIV (number = 24)**	**During SB (number = 24)**	***P*** **value**
TST (minutes)	250 (166 to 330)	98 (42 to 176)	0.0003
Stage N1 (%)	6 (3 to 13)	15 (10 to 26)	0.003
Stage N2 (%)	36 (25 to 52)	49 (23 to 63)	0.6
Stage N3 (%)	38 (12 to 47)	6 (0 to 26)	0.003
REM (%)	13 (3 to 21)	1 (0 to 5)	0.005
Fragmentation (n/h)	26 (14 to 35)	39 (28 to 58)	0.002

Fragmentation, number of arousals and awakenings per hour of sleep. Values are median (25th to 75th percentiles). *P* values from the Wilcoxon rank test. NIV: non-invasive ventilation; REM: rapid eye movement sleep; SB: spontaneous breathing; TST, total sleep time.

Median (25th to 75th) kappa coefficient was 0.68 (0.61 to 0.75) and ranged from 0.44 (reasonable agreement) to 0.78 (good agreement) for pairwise comparison of conventional scoring (n = 24 PSGs).

Intraclass coefficients were 0.90 (0.79 to 0.96) for sleep percentage spent in sleep bouts, 0.82 (0.62 to 0.92) for sleep percentage spent in short naps and 0.91 (0.80 to 0.96) for sleep percentage spent in long naps. Scorings with the lower interrater agreement were also the scorings displaying the larger differences between sleep continuity metrics (see Additional file 2).

## Discussion

The primary objective of this study was to search for pertinent quantification of disrupted hypnograms in ICU patients and to perform an advanced evaluation of this new metric. Using a group of patients treated with NIV for respiratory failure, our results demonstrated that patients with NIV success had high sleep continuity, that is, they spent a substantial proportion of their sleep in naps lasting more than 10 minutes rather than in very short sleep bouts. Conversely, patients with NIV failure had low sleep continuity, that is, they spent a high proportion of their sleep in sleep bouts rather than in naps lasing more than 10 minutes. On the other hand, conventional sleep metrics, such as percentage of sleep stages and the Arousal/Awakening Index, did not significantly differ between groups. Our results also showed that NIV had improved sleep continuity by reducing the proportion of time spent in sleep bouts and by increasing the proportion of time spent in long naps. Finally, sleep continuity has a high reproducibility across scorers.

Most patients in ICUs experience severe sleep disruptions including decreased total sleep time and slow wave sleep, decreased REM sleep and marked sleep fragmentation [1-6]. Sleep fragmentation has captured attention because this index is well-correlated to impaired behavioral performance the day after a fragmented night in healthy subjects [26,27] and because searching for events occurring in the seconds preceding arousal might facilitate the identification of its cause [5]. However, no studies have demonstrated a significant association between sleep fragmentation and outcome in ICU patients. Our results show that arousal indices cannot distinguish patients with NIV success from those with NIV failure. These findings are in line with the study by Trompeo *et al*., which showed a similar fragmentation index in patients with high and low clinical severity scores [28].

As an alternative to arousal counting, several studies have shown that sleep continuity may provide a pertinent approach allowing quantification of sleep structure and quality [17,18,29-33]. To our knowledge, our work is the first study to investigate sleep continuity in critically ill patients. Our results are in line with previous studies in patients showing that measurement of sleep continuity can reveal substantial differences between patients with or without obstructive sleep apnea, differences that are not significant when comparing arousal indexes [17,18]. Our results showed that, during NIV, patients spent less time in bouts and more time in long naps; these findings are congruent with the higher amount of slow wave sleep and REM sleep during NIV. This is in line with the hypothesis that a minimal amount of light sleep continuity is necessary before sleep deepens to N3 and cycles to REM sleep [30,34]. Our findings suggest that, rather than arousal indices, percentage of time spent in sleep episodes lasting less than 10 minutes might be a relevant indicator of sleep alteration in ICU patients. Replication of our findings on a larger group is, nevertheless, required.

Although prolonged PSG, including a large part of the daytime period, is the reference standard for quantifying sleep [35], several sources of bias may have weakened our results. First, we did not record continuously present environmental stimuli such as noise, light and caretaking activities, and different rates of sleep interruption between the two groups cannot be excluded. Although sleep fragmentation indices were similar in patients with NIV success and NIV failure, it is imaginable that environmental factors had more impact on sleep continuity than on fragmentation. However, these environmental factors account for only 30% of awakenings in ICU patients [4,5]. Moreover, we did not assess circadian rhythms in our patients. Critically ill patients often exhibit a disruption of circadian rhythmicity, and low sleep continuity could be a consequence of circadian disruption and/or misalignment [36,37]. A second caveat is the use of 17-hour recordings, instead of 24-hour PSG with a gap from 9 am to 4 pm. Several studies have emphasized the importance of 24-hour recordings in the ICU [36,37]. Recordings of patients with altered distribution of sleep (that is, more sleep during daytime) could therefore be misinterpreted by the sleep continuity metric. It seems possible that in the hours between 9 am and 4 pm, patients experience sleep/wake patterns differently, depending on their mode of ventilation or degree of disturbance of their circadian time-keeping. Nursing care could also have favored one type of sleep bout and then interfered with our findings. Our results need to be confirmed using 24-hour recordings and a time-of-day effect on sleep continuity needs to be explored. Thirdly, we quantified sleep apnea using thoracic and abdominal inductance plethysmography signals rather than oronasal airflow. Inductance plethysmography has been found reliable in quantification of breath waveforms [38]. Although the apnea-hypopnea index did not differ between the groups in our study, we cannot rule out the possibility that the patients with lower sleep continuity had severe sleep disruption due to sleep apnea. The fact that sleep continuity is likely to be influenced by a variety of processes ranging from mechanical ventilation and environmental stimuli to the endogenous circadian rhythm adds to its appeal as a physiologic measure of sleep.

Finally, although our results seem promising, we must underline that they originate from a subgroup of ICU patients. The generalization of our results to other ICU patients appears premature. The investigation of sleep continuity measurement on a larger group, including patients with various degrees of severity of illness and various pathologies, needs to be conducted before extended use of our measures can be envisioned.

Our results show that patients with NIV success spent more time in short naps lasting 10 to 30 minutes and less time in sleep bouts. Numerous studies have demonstrated the beneficial effects on behavioral performance of diurnal naps following sleep restriction or sleep deprivation [21-24]. Our findings are consistent with the sleep continuity theory of Bonnet, which posits that at least 10 minutes of uninterrupted sleep are needed to serve a recuperative function [16,39]. Numerous studies in sleep-deprived healthy subjects have demonstrated that 10- to 20-minute naps exerted a favorable influence with regard to various performance measures [21-24]. Further work is mandatory to transpose Bonnet’s theory from sleep continuity’s effects on vigilance in healthy volunteers to sleep continuity’s beneficial effects on respiratory performance in ICU patients.

## Conclusions

Our work shows that sleep continuity measurements may provide clinically significant data, and be complementary to conventional analysis. Given the high interscorer reproducibility of this method, these measurements could facilitate the sharing of sleep quantification across ICUs. Sleep continuity might be a useful measure of sleep quality in all patients in whom the distribution of sleep episode duration could be reliably determined; sleep continuity could be calculated using the automated EEG analysis recently reported in ICU patients [40]. However, before being considered as an effective alternative metric in the study of sleep in ICUs, sleep continuity measurements need to be replicated and tested in critically ill patients with atypical sleep, in patients with a high severity of illness and in patients receiving sedatives.

## Key messages

- Measurement of sleep continuity may be
  - a clinically significant quantification of sleep,
  - complementary to conventional analysis in ICU patients with normal EEG patterns.
- Patients with NIV success spent
  - a higher proportion of their sleep in naps longer than 10 minutes
  - and a lower amount of sleep in very short sleep bouts (shorter than 10 minutes), than patients who had failed NIV.
- Measurement of sleep continuity
  - shows a high inter-scorer reproducibility
  - could facilitate the sharing of sleep quantification across ICUs.

## Additional files

Additional file 1:
**Flow chart of Study 2. Diagram illustrates patient selection in study 2. NIV: non invasive ventilation.**
Additional file 2:
**Relationship between Cohen’s kappa coefficient and interscorers’ difference in sleep continuity parameters.** Cohen’s kappa coefficient. Each plot represents one PSG. Note that differences between scorers increase along with the diminution of the kappa coefficient.

## Abbreviations

AASM: American Academy of Sleep Medicine
EEG: electroencephalogram
ICC: intraclass coefficient
N2: N2 sleep stage
N3: N3 sleep stage
NIV: non-invasive ventilation
PSG: polysomnography
REM: rapid eye movement sleep
TST: total sleep time
