# Supplementary material for: Sleep continuity: a new metric to quantify disrupted hypnograms in non-sedated intensive care unit patients
Source: Crit Care. 2014 Nov 25;18(6):628. doi: 10.1186/s13054-014-0628-4 (PMC4271438; doi:10.1186/s13054-014-0628-4)
Supplement: Additional file 1: — Flow chart of Study 2. Diagram illustrates patient selection in study 2. NIV: non invasive ventilation. [file 13054_2014_628_MOESM1_ESM.pdf]

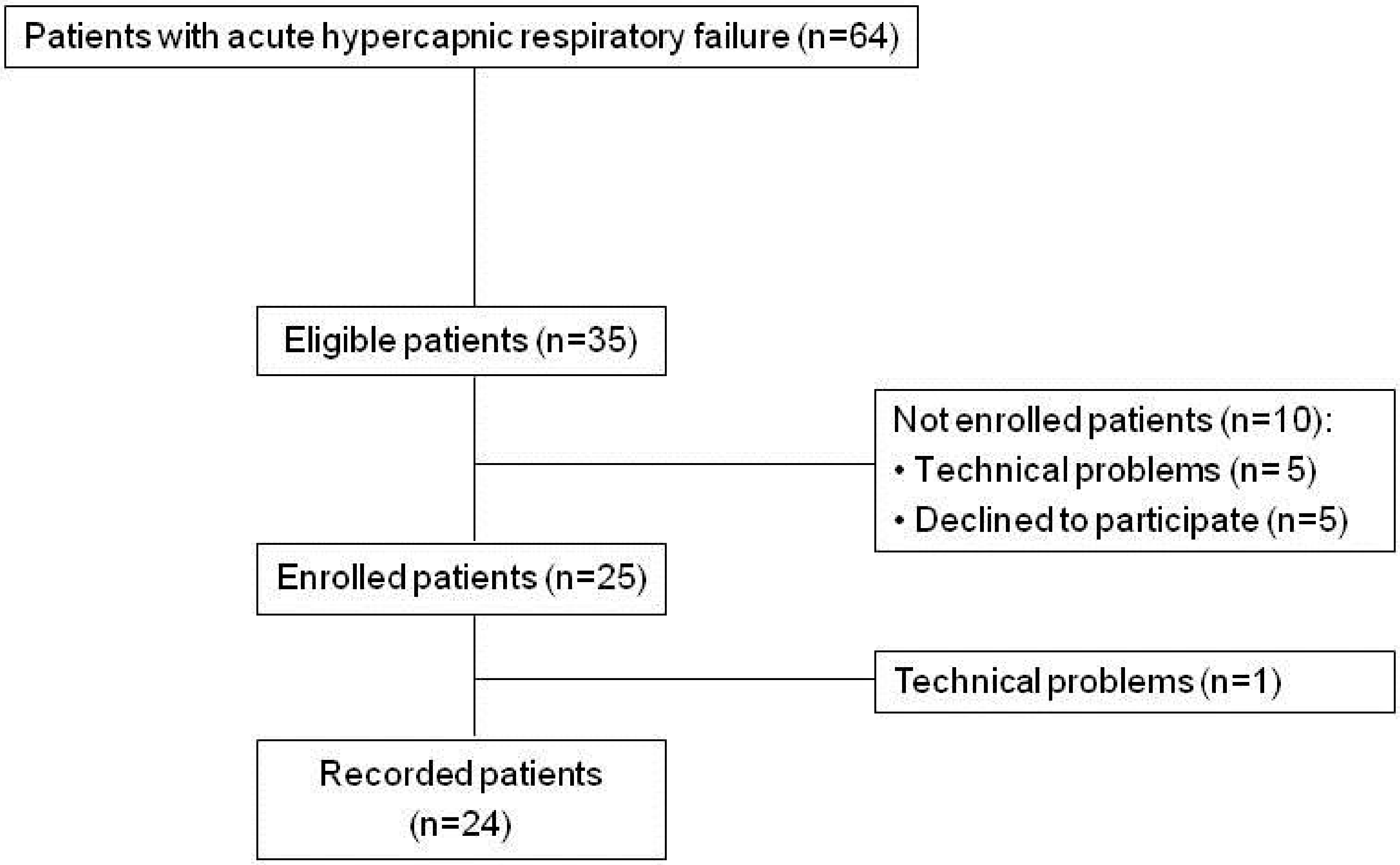
Patients with acute hypercapnic respiratory failure (n=64)
Eligible patients (n=35)
Not enrolled patients (n=10):
• Technical problems (n= 5)
• Declined to participate (n=5)
Enrolled patients (n=25)
Technical problems (n=1)
Recorded patients
(n=24)
