# Supplementary figures and images for: Sleep continuity: a new metric to quantify disrupted hypnograms in non-sedated intensive care unit patients
Source: Crit Care. 2014 Nov 25;18(6):628. doi: 10.1186/s13054-014-0628-4 (PMC4271438; doi:10.1186/s13054-014-0628-4)

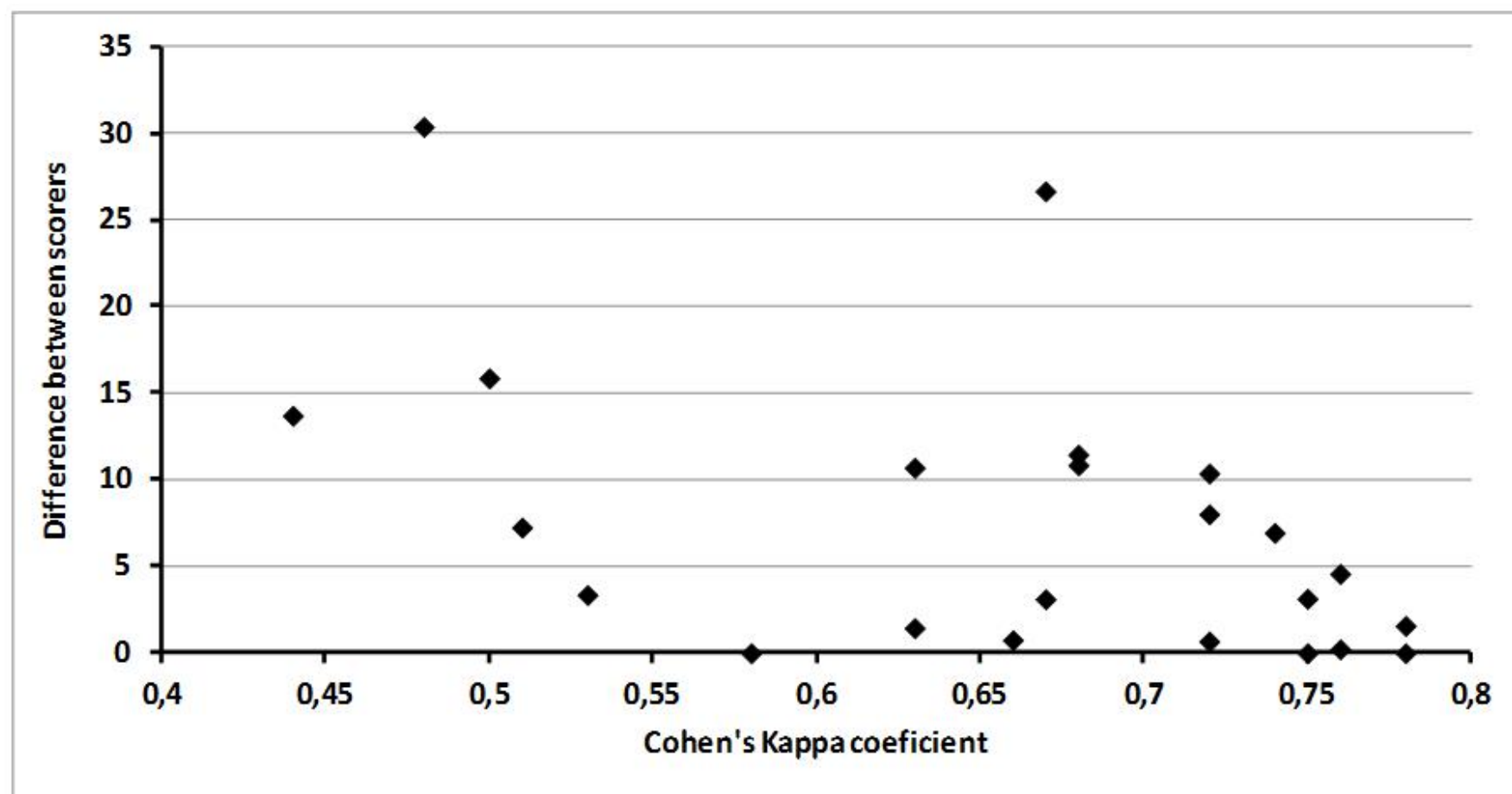

Supplement: Additional file 2: — Relationship between Cohen’s kappa coefficient and interscorers’ difference in sleep continuity parameters. Cohen’s kappa coefficient. Each plot represents one PSG. Note that differences between scorers increase along with the diminution of the kappa coefficient. [file 13054_2014_628_MOESM2_ESM.pdf]
